# Hypofractionated versus conventional fractionation external beam radiotherapy in intermediate and high risk localized prostate cancer

**DOI:** 10.1007/s12672-024-00876-7

**Published:** 2024-02-02

**Authors:** Eileen Samuel, Saliha Zaman, Muhammad Abu Bakar, Muhammad Mohsin Fareed

**Affiliations:** 1 Department of Clinical and Radiation Oncology, Shaukat Khanum Memorial Cancer Hospital and Research Center, Lahore, Pakistan; 2 Lahore Medicare Hospital, Lahore, Pakistan; 3 Cancer Registry and Clinical Data Management, Shaukat Khanum Memorial Cancer Hospital and Research Center, Lahore, Pakistan; 4 Department of Radiation Oncology, West Virginia University School of Medicine, 44 Medical Center Drive, Morgantown, WV 26505 USA

**Keywords:** Hypofractionated radiotherapy, Conventional radiotherapy, Prostate cancer, Intermediate and high risk prostate cancer, Proctitis, Cystitis

## Abstract

**Background:**

Prostate cancer is the second most common malignancy in men, and its incidence is increasing which is attributed to increased screening programs. The treatment options of intermediate and high risk prostate cancer include radical prostatectomy, radiotherapy and androgen deprivation therapy. Hypofractionated radiotherapy is becoming more popular lately due to better understanding of the radiobiology of prostate cancer and favorable logistics.

**Objective:**

To compare the toxicity and efficacy of hypofractionated versus conventional fractionation external beam radiotherapy in patients with intermediate and high risk localized prostate cancer treated in Shaukat Khanum Memorial Hospital and Research Center, Lahore (SKMCH & RC).

**Methodology:**

We retrospectively conducted this study on histopathologically confirmed 114 patients with prostate adenocarcinoma who underwent treatment from January 2013 till December 2018. These patients were treated with radical radiotherapy along with hormonal therapy as per indication. Data was collected from electronic hospital system and analyzed by SPSS version 23.

**Results:**

114 patients were selected according to the inclusion criteria. Mean age was 68 years (61–75). 88% of patients had stage III-IVA disease at the time of diagnosis. Mean PSA and GS was 33 ± 39 SD and 7 ± 0.9 SD respectively. 89% (n = 102) received radiotherapy with 69% of patients receiving dose of 60 Gy in 20 fractions. Among patients who received hypofractionated dose, 86% (n = 61) of them were categorized as high risk and 14% (n = 10) were intermediate risk, whereas among conventional group 90% (n = 28) were high risk patients and 10% (n = 3) were of intermediate risk. In hypofractionated dose group, 14% (n = 10) developed grade 2 proctitis and 8% (n = 6) developed grade 2 cystitis, in contrast to conventional dose group in which only 3 patients (5%) developed grade 2 GI toxicity and 2 patients (2.9%) had grade 2 GU toxicity. However, these toxicities and their grade were clinically insignificant when compared with the dose groups (p = 0.11). 5 year overall survival for hypofractionated radiotherapy versus conventional dose was 100% and 90% respectively with 95% Cl and p value of 0.3 (clinically insignificant), whereas 5 year disease free survival was 100% and 75% for hypofractionation versus conventional EBRT respectively with 95% CI and p value of 0.04 (clinically significant).

**Conclusion:**

Hypofractionated radiotherapy in patients with intermediate and high risk localized prostate cancer has better disease free survival at the expense of higher risk for proctitis and cystitis but no difference in overall survival as compared to conventional dose of radiation.

## Introduction

The incidence of prostate cancer is considerably higher in the Western world when compared with the East; however, the overall burden of cancer patients is 60% in East versus only 20% in Western population [1]. World Health Organization (WHO) estimated the mortality rate of cancer to be nearly 70% in developing countries [2, 3]. According to GLOBOCAN report in 2018 [1], the incidence of prostate cancer was 18.1 million, hence constituting 7.1% of total cancer cases. During the same year, a meta-analysis conducted in Pakistan revealed the prevalence of prostate cancer to be 5% (range 2–8%) [4], however the average risk in men for the development of microscopic disease, clinical disease and case specific mortality rate are 30%, 10% and 3% respectively [5] for prostate cancer.

Prostate cancer (PCa) is the second most common cancer and the fifth leading cause of death among men [6]. It is postulated that hereditary PCa constitute less than 5%. However, the risk is increased by a factor of 1.3 or 2.5 if father or brother had PCa respectively [7]. PCa is often asymptomatic in the initial stages, as it starts in the posterior lobe of the gland but can be easily palpable on digital rectal examination (DRE). It usually progresses very slowly but when it compresses the urethra patient may complain of polyuria, weak stream with hesitancy in urination, dysuria, hematuria or may experience erectile dysfunction. Diagnostic investigations include prostate specific antigen (PSA), Gleason score (GS), MRI of pelvis, biopsy via transurethral ultrasound (TRUS) or transurethral resection of prostate (TRUP) and bone scan.

The treatment of prostate cancer includes radical prostatectomy, external beam radiotherapy (EBRT via conventional or hypo-fractionation), brachytherapy, chemotherapy and androgen deprivation therapy. Previous studies have demonstrated that combination of androgen deprivation therapy (ADT) with a primary treatment (radical prostatectomy or radiotherapy) can locally control the disease more effectively when compared with either single modality treatment [8–10]. This is because ADT has an effect on the tumor’s genetic expression and potentiates apoptosis, hence can be used to treat local and distant disease [10]. However, life expectancy depends on several factors. These include age, co-morbidities, grade of tumor, GS, PSA, disease status, time to receive treatment and response to treatment.

Historically, conventional radiation dose ranging from 70 to 81 Gy was recommended for patients of prostate cancer depending on the risk stratification [11–13]. However, this dose range and fractionation protract a lengthy treatment time, higher risk for adverse effects and eventually yield lower treatment efficacy. There is sufficient evidence to suggest that due to low α/β ratio for PCa, a dose range from 0.9 to 2.2 Gy per fraction is effective for its treatment [14]. Similarly, there is radiobiologic advantage that hypofractionated treatment dose could enhance treatment effects [15], as a dose of ≥ 2.5 Gy per fraction could theoretically maintain high biologically effective doses, while not increasing acute and late adverse events and efficiently shortening the treatment time. Such outcomes would translate into higher treatment capacity and could potentially reduce treatment cost [16].

### Objective

To compare the side effects and efficacy of hypofractionated (2.4–3.0 Gy/fraction) versus conventional (1.8–2.0 Gy/fraction) fractional external beam radiotherapy in patients with intermediate and high risk localized prostate cancer treated at Shaukat Khanum Memorial Hospital and Research Center, Lahore.

## Materials and methods

Ethical approval was waived by the local Ethics Committee of Shaukat Khanum Memorial Cancer Hospital & Research Center in view of the retrospective nature of the study and all the procedures being performed were part of the routine care. Informed consent was Waived from all subjects and/or their legal guardian(s). 114 patients without age limit were selected from database set of hospital system who had histopathologically proven adenocarcinoma of prostate cancer, had locally advanced disease on MRI guided staging and underwent radical treatment from Shaukat Khanum Memorial Hospital from January 2013 till December 2018. Biopsies and transurethral resection of prostate were permitted. Patients treated with radical prostatectomy and patients with either low grade prostate cancer or metastatic prostate cancer at the time of presentation or after receiving radical treatment were excluded from the study. These patients were treated with either hypofractionated dose or conventional dose of radiotherapy along with 2 year hormonal therapy as per hospital protocol. All methods were performed in accordance with the relevant guidelines and regulations. The margins for PTV for both the groups were 5–10 mm in all directions, except in hypofractionated arm, where it was 3–5 mm posteriorly. Common Terminology Criteria for Adverse Events (CTCAE) was used for assessment and grading of toxicities. PSA of all these patients was then followed for 5 years for identification of biochemical failure.

## Data collection and analysis

Data was collected from standardized electronic medical record and then analyzed by SPSS version 23. Data was stratified for age, risk assessment (PSA, GS, stage), initial PSA, stage of disease, duration and intent of treatment, dose and fractionation of radiotherapy, response of treatment and toxicities to address effect modifiers. Mean ± standard deviation was used for quantitative data like age, initial PSA, dose and fractionation of radiotherapy and time for response of treatment. All qualitative variables like risk assessment, stage of disease, outcome of treatment and grades of toxicities were presented in the form of frequency (%). Kaplan Meier survival curve was used for drawing overall and disease free progression survival with confidence interval of 95% calculated for clinical outcomes and P value of ≤ 0.05 was considered significant.

## Results

**Fig. 1:**
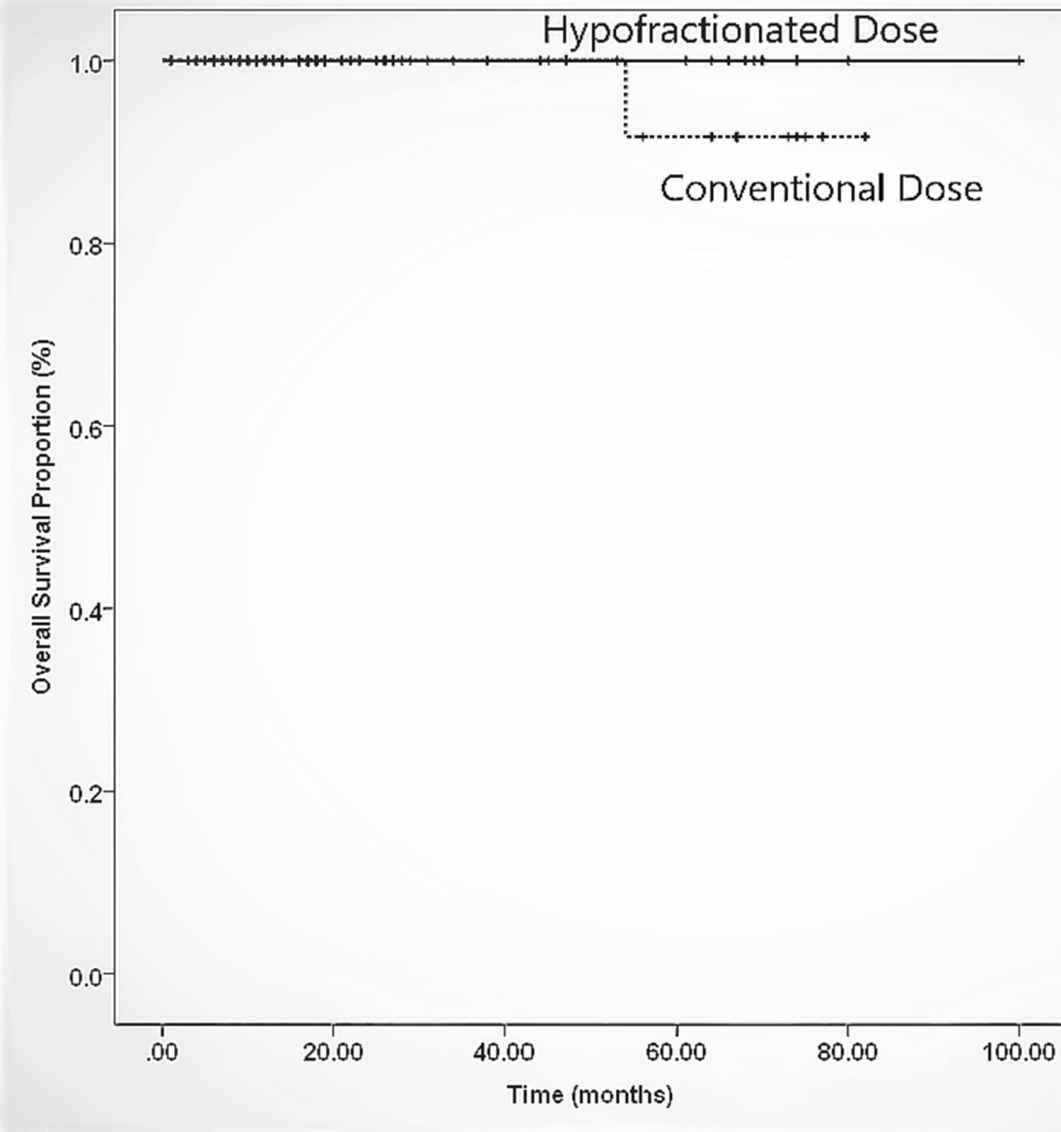
Overall survival of patients receiving hypofractionated versus conventional dose

**Fig. 2:**
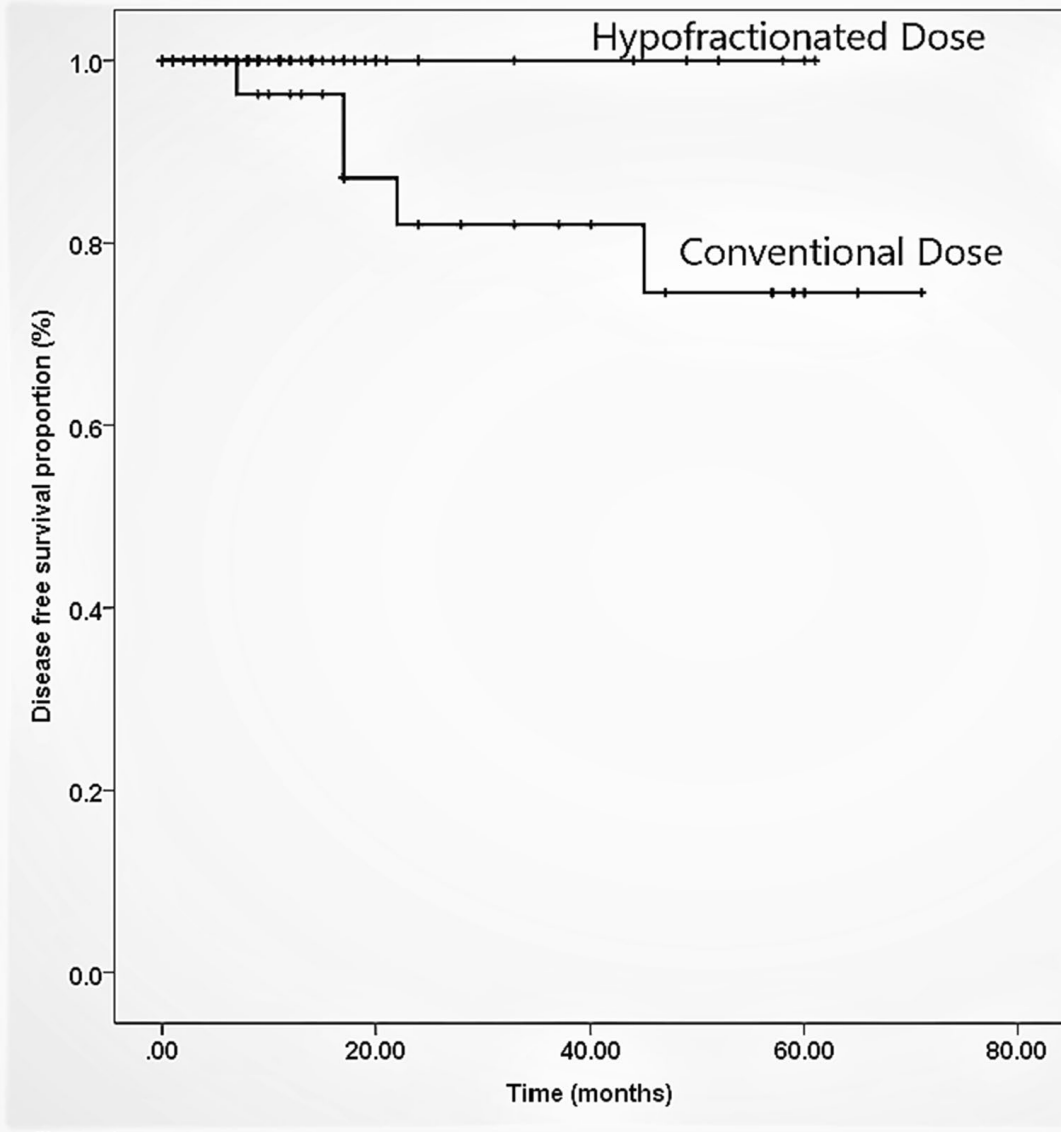
Disease free survival of patients receiving hypofractionated versus conventional dose

Our study showed that out of total 114 patients 12% (n = 14) had an age range of 48–60 years, 56% (n = 64) were between 61 and 70 years and 32% (n = 36) were older than 71 years. Mean age was 68 years (61–75) and 93.4% had ECOG PS of 0–1, 88% of patients had stage III-IVA disease at the time of diagnosis with only 21% (n = 24) with nodal involvement. Mean PSA was 33 ± 39 SD while mean GS was 7 ± 0.9 SD. 68% patients received ADT for ≤ 2 years. Furthermore, 89% (n = 102) received radiotherapy with 69% of patients receiving dose of 60 Gy in 20 fractions while 31% got 70–76 Gy in 28–38 fractions. Among patients who received hypofractionated dose, 86% (n = 61) of them were categorized as high risk and 14% (n = 10) were intermediate risk according to NCCN guidelines whereas among conventional group 90% (n = 28) were high risk patients and 10% (n = 3) were of intermediate risk. Table 1 is displaying patient characteristics. The patients who received hypofractionated dose, 14% (n = 10) developed grade 2 proctitis and 8% (n = 6) developed grade 2 cystitis. In contrast, those who received conventional dose, only 3 patients (5%) developed grade 2 GI toxicity and 2 patients (2.9%) had grade 2 GU toxicity (Table 2). None of the patients developed G 3-5 toxicity.  However, these toxicities and their grades were clinically insignificant when compared with the dose groups (overall p = 0.11 and specifically for proctitis and cystitis were 0.729 and 0.252 respectively). 10% patients (n = 11) had biochemical failure while 12% (n = 14) developed distal metastasis. 5 year overall survival for hypofractionated radiotherapy versus conventional dose was 100% and 90% (Fig. 1) respectively with 95% Cl and p value of 0.3 (clinically insignificant), whereas 5 year disease free survival was 100% and 75% for hypofractionation versus conventional EBRT (Fig. 2) respectively with 95% CI and p value of 0.04 (clinically significant).

**Table 1:** Patient characteristics

	Hypofractionation (69%)	Conventional fractionation (31%)
Age (yrs)	67 ± 6.5 SD	69 ± 6 SD
Stage (TNM)		
I	1.4%	0%
II	30%	0%
IIIA	15.7%	40.6 %
IIIB	37%	40.6 %
IVA	15.7%	18.8%
PSA	33 ± 43 SD	31.6 ± 29 SD
GS	7.3 ± 0.96 SD	7.5 ± 1 SD
NCCN risk group		
Intermediate	14%	10%
High	86%	90%
ADT		
≤ 1 year	24%	18.8%
1–2 years	33%	6.3%
≥ 2 years	41%	71%

**Table 2:** Comparison of CTCAE toxicities among hypofractionation versus conventional fractionation

CTCEA toxicities	Hypofractionation (69%)	Conventional fractionation (31%)
Proctitis		
Grade I	6.4	8.3
Grade II	14	5
Cystitis		
Grade I	31	4.3
Grade II	8	2.9

## Discussion

Our study showed that the mean age at prostate cancer presentation is 68 years ± 7 SD with majority having good performance status even with stage III or node positive disease as prostate cancer is a slow growing tumor. All patients are treated with combination treatment of ADT with radiotherapy. However, our study showed that hypofractionation improve DFS but did not show any difference in OS. Longer duration studies and more randomized trials are required to further elaborate this, as these patients typically do not die of prostate cancer. With advent of improved radiotherapy technologies and image guidance, hypofractionated EBRT plays a crucial role in the treatment of intermediate to high risk PCa as it requires shorter duration of treatment with equal efficacy in OS and superiority in terms of DFS when compared with conventional radiation dose. Although hypofractionated radiotherapy did not significantly improve overall survival, it enhanced biological efficacy of delivered radiation dose and reduced overall treatment time, presumably making the treatment more acceptable for patients. This explains clinically significant difference in DSF detected between groups. 10% of our patients had biochemical failure while 12% developed distal metastasis treated with either conventional or hypofractionated EBRT. The patients who received hypofractionated dose in our study, 14% (n = 10) of them suffered from CTCAE (Common Terminology Criteria for Adverse Effects) grade 2 proctitis and 8% (n = 6) developed grade 2 cystitis, whereas none of treated patients experienced grade 3 or more GI/GU toxicities. In comparison, those who received conventional dose, only 3 patients (8%) developed grade 2 GI toxicities and 2 patients (2.9%) had grade 2 GU toxicities which is approximately one third of toxicities seen in patients receiving hypofractionated dose. However, no QOL assessments questionnaires were done for further evaluation.

Meta analysis by Guo et al. [17] showed that the efficacy and adverse effects were similar for hypofractionated and conventional radiotherapy in the treatment of intermediate to high risk localized prostate cancer [17]. This analysis illustrated that OS (HR = 1.12, 95% CI 0.93–1.35, *p* = 0.219) and prostate cancer specific survival (HR = 1.29, 95% CI 0.42–3.95, *p* = 0.661) were similar in both treatment groups. Furthermore, acute GI toxicity was higher in hypofractionated group (RR = 1.70, 95% CI 1.12–2.56, *p* = 0.012) and late GI effects were more in conventional treatment group (RR = 0.75, 95% CI 0.61–0.91, *p* = 0.003), however acute (RR = 1.01, 95% CI 0.89–1.15, *p* = 0.894) and late GU toxicities (RR = 0.98, 95% CI 0.86–1.10, *p* = 0.692) were same in both groups. Another study conducted by Wilkin et al. also demonstrated that hypofractionated radiotherapy is non inferior to conventional radiotherapy when the incidence of patient reported bowel symptoms were compared after 24 months of irradiation in both groups [18]. Moderate hypofractionation was also found to be safe and effective in localized prostate patients even with oligometastatic disease [19]. A randomized, multicenter, phase III, Hypofractionation irradiation for prostate cancer (HYPRO) trial was conducted from 2007 to 2010 in intermediate and high risk prostate cancer. Comparable relapse free survival was noted with hypofractionation treatment [20]. Furthermore, at 7 years, the reported update suggested that hypofractionation is neither superior nor inferior to the conventional therapy [21]. When toxicities were compared in this trial, initial results did not reveal any significant differences in grade 2 or higher late toxic effects between the two groups [22, 23]. In 2018 while assessing the quality of life of these patients, results showed that sexual dysfunction related to ADT hypofractionation was found to be non- inferior to conventional fractionation [24]. Numerous randomized trials have also shown that the adverse events of hypofractionated radiotherapy were similar to conventional radiotherapy [25–28].

National Cancer Data Base study showed that escalated radiotherapy dose resulted in an improvement in OS for patients with intermediate to high risk PCa [29]. Similarly, Kuban et al. [30] published their data of 301 patients with T1b to T3 PCa showing that 78 Gy is superior in maintaining biochemical failure when compared to 70 Gy (78% vs. 59%, *p* = 0.004). However, conventionally delivered fractionation had higher risk for increased toxicity.

One of the limitations of our study was being not able to calculate hazard ratio and relative risk ratio for either group, due to low median follow up of only 5 years. Inherently, majority of the patients belonged to high risk category. For these to be analyzed, further larger, randomized trials need to be conducted for 9–10 years by which further queries for OS will be clarified on dose selection as prostate cancer has indolent nature. Long term follow up is of special significance to assess differences in overall survival [31].

## Conculsion

For localized intermediate and high risk prostate cancer patients, hypofractionated course of EBRT results in better disease free survival at the expense of higher G-2 proctitis and cystitis. No difference was seen in overall survival between hypofractionated radiotherapy and conventional radiation dose. Randomized trials with longer follow up and larger sample size are required to elaborate this difference further.

## Data Availability

The data that support the findings of this study are available from the authors but restrictions apply to the availability of these data. Data are, however, available from the authors upon reasonable request and with permission from the Shaukat Khanum Memorial Cancer Hospital & Research center repository.
